# Improving Support for Family Caregivers of People with a Serious Illness in the United States: Strategic Agenda and Call to Action

**DOI:** 10.1089/pmr.2020.0004

**Published:** 2020-04-30

**Authors:** Peter Hudson, R. Sean Morrison, Richard Schulz, Abraham Aizer Brody, Constance Dahlin, Kathleen Kelly, Diane E. Meier

**Affiliations:** ^1^ Centre for Palliative Care, St Vincent's Hospital, Melbourne, Australia.; ^2^ The University of Melbourne, Melbourne, Australia.; ^3^ Vrije University Brussels, Belgium.; ^4^ National Palliative Care Research Center, Icahn School of Medicine of Mount Sinai, New York, New York, USA.; ^5^ Brookdale Department of Geriatrics and Palliative Medicine, Icahn School of Medicine of Mount Sinai, New York, New York, USA.; ^6^ Lilian and Benjamin Hertzberg Palliative Care Institute, Mount Sinai Hospital, New York, New York, USA.; ^7^ Center for Social and Urban Research, Education, and Policy, University of Pittsburgh, Pittsburgh, Pennsylvania, USA.; ^8^ Department of Psychiatry, School of Medicine, Education, and Policy, University of Pittsburgh, Pittsburgh, Pennsylvania, USA.; ^9^ Center for Caregiving Research, Education, and Policy, University of Pittsburgh, Pittsburgh, Pennsylvania, USA.; ^10^ Rory Meyers College of Nursing, New York University, New York, New York, USA.; ^11^ Hartford Institute for Geriatric Nursing, New York University, New York, New York, USA.; ^12^ Center to Advance Palliative Care, New York, New York, USA.; ^13^ Hospice and Palliative Nurses Association, Pittsburgh, Pennsylvania, USA.; ^14^ North Shore Medical Center, Salem, Massachusetts, USA.; ^15^ Family Caregiver Alliance, National Center on Caregiving, San Francisco, California, USA.; ^16^ Patty and Jay Baker National Palliative Care Center, Icahn School of Medicine of Mount Sinai, New York, New York, USA.; ^17^ Department of Geriatrics and Palliative Medicine, Icahn School of Medicine of Mount Sinai, New York, New York, USA.; ^18^ Medical Ethics, Icahn School of Medicine of Mount Sinai, New York, New York, USA.

**Keywords:** family caregivers, palliative care, policy, serious illness

## Abstract

***Background:*** An estimated 30% of the adult American population are caregivers and many of the people they support live with serious illnesses. Caregivers provide an average of 20 hours of services per week and are heavily involved in assisting with activities of daily living. This input represents considerable economic value to the health care system and to the well-being of communities. However, the impact of the burden on caregivers is considerable with negative outcomes on their physical, psychological, social, and financial well-being. The current landscape of caregiver policy in the United States is not well coordinated and does not meet the needs of this population.

***Objective:*** To develop a strategy to enhance the future of family caregiver support of people with serious illness within the United States.

***Methods:*** (1) Creation of project steering and key stakeholder groups; (2) survey and in-depth interviews with key stakeholders; (3) review of key family caregiver reports, systematic reviews, policies, and financial initiatives.

***Results:*** A strategy to provide clear direction to enhance the future of family caregiver support of people with serious illness within the United States was developed focusing explicitly on policy, research, training, service delivery, and public engagement.

***Conclusions:*** The strategy is an initial step aimed at enhancing support for family caregivers of people living with serious illness. It outlines key recommendations and a “call to action.” Subsequent work will be needed on prioritization of tasks, gaining buy-in at all levels of the policy-making apparatus, operationalization, and implementation.

## Background

An estimated 66 million Americans, or ∼30% of the adult population, are caregivers and nearly two-thirds are women. They provide an average of 20 hours of services per week and are heavily involved in assisting with instrumental activities of daily living.^1^ Although some family members may readily assume this responsibility, many others do so based on a sense of duty or obligation. The impact of the role increases when family caregivers must administer medications (including opioids); maintain complex equipment; and perform the physical labor of feeding, bathing, toileting, changing and cleaning, dressing, turning, and transporting a family member. The psychological burden is exacerbated when caregivers worry about performing all those tasks safely, when caregiving keeps them from meeting responsibilities to other family members, when the person being cared for is frightened or in pain, and when they (the caregivers) receive little training or guidance.^1^ Caregiving also takes a financial toll when families face high out-of-pocket costs for services and equipment or when family income decreases because family caregivers must reduce their work hours or leave their jobs altogether.^1^ In addition, >70% of patients with serious illness in the United States receive care from family caregivers at the time of their death, and an estimated three million family caregivers accompany patients through the dying process annually.^2^

The care provided by family caregivers represents considerable economic value to the health care system and to the well-being of communities.^2^ However, in today's world, family caregivers cannot be expected to provide complex care and support on their own. Family caregivers need greater recognition, information, and meaningful support to help them care for older relatives or friends, and to maintain their own health, financial security, and well-being.^3^ Given the global impact of COVID-19, caregiving tasks are increasingly being conducted remotely (and independently), thus increasing the urgency of a coordinated and systematic response.

The current landscape of caregiver policy is a patchwork of small uncoordinated programs that do not yet meet the needs of this population.^4^ Although the United States spends significantly more than other nations in health care, it spends relatively little in comparison on social support.

The implications of these inadequacies are particularly problematic for family caregivers of patients with a serious illness; for example, those affected by multiple chronic illnesses, dementia, or at end of life.^5^ There is no systematic approach in place to assess and respond to the needs of family caregivers of people with serious illness. National practice guidelines in the United States identify family caregiver support for people with a serious illness as an essential element of palliative care provision.^6^ However, palliative care services are not equipped to meet all of the ongoing needs of caregivers of people living with a serious illness and it is unlikely that even with increased resources, they would be able to do so. Therefore, any strategy to enhance caregiver support in the United States must also involve other key stakeholder groups.

### Project purpose and scope

This project aimed to develop a strategy to provide clear direction to enhance the future of family caregiver support of people with serious illness within the United States. The strategy is targeted at policy makers, clinicians, payers, employers, and service providers who are involved in the care of people with serious illness. The strategy is presented in a relatively succinct “high level” format and should be viewed as an initial phase where key recommendations are put forward. Subsequent work will be needed on prioritization of tasks (including identifying actors responsible), operationalization, and implementation. We also acknowledge that some of the recommendations are not exclusively applicable to family caregivers of patients with serious illness.

## Methods

The process to develop the strategy comprised the following steps:

1. Establishment of a project steering group to help guide the project, comprising the Director (D.E.M.) of the U.S. Center to Advance Palliative Care; the Director (R.S.M.) of the U.S. National Palliative Care Research Center, and a leading U.S. palliative nursing academic (A.A.B.) from New York University.
2. Brief survey of key stakeholders from across the United States that incorporated open-ended questions about (a) the main barriers and enablers to effective family caregiver support, (b) identifying seminal caregiver reports and systematic reviews, (c) insights into the priority ingredients for a strategy, (d) other key stakeholders to approach for input, and (e) willingness to be involved in an in-depth interview. Thirty stakeholders were identified from the steering group, including representatives from national caregiver groups, policy makers, palliative care clinicians, and academics. Twenty stakeholders completed the survey.
3. In-depth interviewing (through telephone, videoconferencing, and face to face where feasible) was conducted with 14 key informants from across the United States to gather their insights into the main elements for a successful strategy. Representatives included 10 of the participants arising from the survey response as well as 4 others who were recommended by the survey participants.
4. Reviewing published literature focusing on seminal U.S. family caregiver-related reports^1,3,4,7^ and a select number of recent family caregiver systematic reviews.^2,8–14^
5. Review of existing family caregiver-related policies and financial initiatives (refer Table 1).
6. Developing a draft strategy to improve family caregiver support based on the preceding steps.
7. Presenting the draft strategy to the key stakeholder group (formed from survey and interview participants) for feedback and refinement.
8. Production of final version of the strategy.

**Table 1.** Examples of Existing U.S. Policies and Financial Initiatives

*The Family and Medical Leave Act* of 1993 guarantees up to 12 workweeks of unpaid leave to each leave year to qualifying employees for specified family and medical leave reasons and, pursuant to amendments to the law, up to 26 workweeks of leave in a single 12-month period to care for a seriously ill or injured covered service member.
*The National Family Caregiver Support Program*, established in 2000 under Title III-E of the Older Americans Act, provides grants to States and Territories, based on their share of the population aged 70 years and over, to fund a range of supports that assist family and informal caregivers to care for their loved ones at home for as long as possible. Individuals served include adults (>18 years) caring for a family member who is either 60 years or older or has Alzheimer's disease and related disorders and cognitive dysfunction. The law requires the state to give priority to elderly individuals with cognitive problems.
*The Lifespan Respite Care Act* of 2006 aimed to create a system of accessible community-based respite care services for family caregivers of children or adults with special needs. Each state Lifespan Respite Care Program involves a partnership between a state agency, a state respite coalition, and an ADRC. The ADRC serves as a single point of entry for individuals seeking long-term care services and administers certain aspects of the state's long-term care system. The ARDC and the state agency may be a single integrated entity.
Many *Medicaid home and community-based service* waivers contain a “self-” or “participant-directed” component that allows the waiver recipient to select and pay their own caregivers, including family caregivers.
*Caregiver Advise, Record, Enable Act* mandates that before hospital discharge, a family caregiver is identified and provided with relevant training to foster optimal discharge planning.
*Acts under consideration:* (S.1028) Recognize, Assist, Include, Support, and Engage Family Caregivers Act of 2017; (S.311) Alzheimer's Caregiver Support Ac; (S.759) Credit for Caring Act.

Adapted from Ref. 4.

ADRC, Aging and Disability Resource Center.

Formal ethics review was not required for this quality improvement initiative. Nonetheless, participants were advised that their contribution was voluntary and their permission was sought to list their names (and associated institutions) as acknowledgments in the final report and any other publications arising from this study.

The following operational definitions were utilized:

*Family caregiver:* Family caregivers (also known as care partners) are relatives, spouses, friends, or neighbors who assist an older adult (referred to in this report as a care recipient) who needs help due to physical, mental, cognitive, or functional limitations. The caregiver's involvement is driven primarily by a personal relationship rather than by financial remuneration. Family caregivers may live with, or apart from, the care recipient. Care may be episodic, or of short or long duration.^3^

*Serious illness:* Serious illness is a condition associated with a high risk of mortality, negatively impacts quality of life and daily function, and/or is burdensome in symptoms, treatments, or caregiver stress.^15^

*Palliative care:* Care that provides relief from pain and other symptoms supports quality of life, and is focused on people with serious advanced illness and their families. Palliative care may begin early in the course of treatment for a serious illness and may be delivered in a number of ways across the continuum of health care settings, including in the home, nursing homes, long-term acute care facilities, acute care hospitals, and outpatient clinics.^3^

## Results

### Strategic goal

The goal of this study was to improve the quality of life of family caregivers of people living with a serious illness in the United States.

### Strategic domains and actions

This project found five key domains (formed from survey and interview participants' data) wherein actions can be initiated to address the significant gaps related to family caregiving for seriously ill older adults: (1) policy and financial; (2) training, education, and translation of evidence; (3) research and evaluation; (4) service delivery; and (5) public engagement.^1^ Hereunder we present bulleted actions that could be implemented under each of these domains to improve the quality of life for family caregivers of people living with serious illness.

#### Policy and financial

- Extend the resources and scope of The National Family Caregiver Support Program (NFCSP), established by the Older Americans Act, which has helped raise awareness of the importance of family caregivers by promoting “the caregiver as a client” and offering family counseling, support groups, training, and respite care.^1^
- Broaden the scope of Medicaid's Cash & Counseling program, available in ∼15 states, which allows beneficiaries to pay family members modest sums for home care services in some cases.^1^
- Ensure the Caregiver Advise, Record, Enable (CARE) Act is extended to all states and that its application and outcomes are comprehensively evaluated and relevant changes made.
- Introduce policy initiatives that enable employers to provide paid caregiver leave. This will require legislative support and endorsement from insurance companies.
- Broaden unpaid leave to other family members: the Family and Medical Leave Act of 1993 guarantees up to 12 weeks (veterans can potentially receive up to 6 months) of job-protected unpaid leave for attending to the care needs of a spouse, child, or parent, but not other family members. Some states have been doing this and going beyond to include paid family leave such as New York State.
- Develop and implement effective mechanisms within Medicare, Medicaid, and the U.S. Department of Veterans that (1) identify caregivers in both the care recipient's and the caregiver's medical record; (2) ascertain family caregivers' needs and those who are at risk; (3) determine types of training and supports they might need to continue their role.^3^
- Include family caregivers in Centers for Medicare and Medicaid Servicespayment and service delivery demonstrations.^3^
- Extend relevant aspects of the Veteran's Affairs family caregiver program to caregivers of non-Veterans.
- Redesign federal, state, and private insurance and health care delivery programs so they integrate the financing of medical and social services to support the provision of quality care consistent with the values, goals, and informed preferences of people with serious illness and their family caregivers.^1^
- Mandate the use of interoperable electronic health records across care services, settings, and institutions that incorporate advance care planning to improve communication of individuals' wishes across time, settings, and providers, and include an assessment and care plan for their family caregiver(s).^1,3^
- Expand tax benefits at the state level, relax the definition of “dependent” and allowance for respite care as an expense under the federal Dependent Care Tax Credit, and increase appropriations to support state Lifespan Respite Care Programs.^4^
- Extend social security credits for caregiving under either the retirement or disability insurance programs.^4^
- Expand employer coverage, eligibility standards under the Family and Medical Leave Act, and incentive grants to states for paid caregiver leave programs.^4^
- Expand the Medicaid Home and Community-Based Services waiver programs to ensure all states mandate an assessment of family caregivers' needs.^3,4^
- Explore, evaluate, and, as warranted, adopt federal policies that provide economic support for working caregivers.^3^ Several states have enacted paid family leave statutes, and/or have paid sick leave laws that require employers to allow workers a reasonable number of earned sick days to care for an ill family member (including some older adults).^3^
- Review Medicare and other advance care planning policies and make relevant amendments related to involvement of family caregivers.
- Develop policies to improve relationships and partnerships between hospital, health care, and community services.
- Create a formal International Classification of Diseases, version 10 (ICD-10) code for caregiver burden to allow health professionals to be reimbursed for providing support to caregivers. Develop also payment mechanisms to foster health providers' engagement with family caregivers even when care recipients are not present.^3^
- Provide financial assistance (where pertinent) for family caregivers to engage an attorney to prepare a plan for the legal and financial implications of being a family caregiver.
- Introduce policy initiatives that ensure employers offer flexible work hours to staff who are also family caregivers.
- Provide financial incentives to providers for implementation of strategies that involve caregiver support (and other supports as pertinent) that decrease the need for emergency room and acute care services, and improve coordination of care across settings and providers.^1^
- Introduce policy initiatives that formally legitimize family caregivers as worthy recipients of health and/or social services.
- Expand reimbursement to community health services for family caregiver support.
- Conduct a critical appraisal of the respite and bereavement services currently provided through the Medicare Hospice Benefit.

#### Training, education, and translation of evidence

- Develop and enforce culturally relevant competencies for identifying, assessing, and supporting family caregivers by health care and human service professionals, together with regulatory and accrediting organizations within the federal government in collaboration with colleges (e.g., American Association of Colleges of Nursing, Association of American Medical Colleges), professional societies, education programs, and licensure and certification bodies.^3^
- Professional societies and other institutions that establish quality standards should develop standards for clinician–patient communication and advance care planning (and related training) that are measurable, actionable, and evidence based^1^ and explicitly involve family caregiver(s).
- The U.S. Department of Health and Human Services (HHS) Office for Civil Rights should clarify caregivers' access to information by providing administrative guidance to health care and social service providers regarding allowable disclosures of protected health information to family caregivers and require providers to educate their workforce regarding that clarification.^3^
- Implement a consensus and evidenced-based national education program (available in a variety of modes) that prepares family caregivers of patients with serious illness for the typical aspects of the role and provides guidance on strategies to respond to the impact(s) of this role.
- Develop a systematic approach to translation and implementation of training for health and social service providers using the three-tiered framework (i.e., individual, organizational, and societal)^3^; also using existing evidence from robust interventions that have shown benefit (for caregiver well-being and/or efficient use of health resources).
- Develop and adopt agreed-upon caregiver assessment tool(s) and associated training required to implement systematic caregiver assessment.^3^
- Perform a formal scoping review and critical appraisal of existing “online” family caregiver resources (e.g., the National Institute of Health [NIH]'s clinical center) to provide a universal national “one stop shop” or virtual national resource center where family caregivers can access evidenced-based easily accessible resources (including information and how to access support).
- Provide employers with guidance and training on best practices to support workers with caregiving responsibilities.^3^
- Provide better training about the Health Insurance Portability and Accountability Act (HIPAA) regulations related to the involvement of family caregivers to foster appropriate information exchange and communication among providers, caregivers, and care recipients.^3^
- Develop information to assist family caregivers when they avail themselves of the Family Medical Leave Act.
- Disseminate best practices of information technology relevant to family caregivers. The Office of the National Coordinator for Health Information Technology is well situated to do this through education and outreach through Regional Extension Centers, through HIT.gov, and by partnering with professional societies and credentialing organizations.^3^
- Ensure education of health and social service providers about current family caregiver support resources and how to access them so they can, in turn, direct family caregivers how to access.
- Engage disease-specific organizations (particularly those diseases that can progress to advanced stage) in the processes of health care professionals' education about family caregiver support.
- Pass the Palliative Care and Hospice Education and Training Act.

#### Research and evaluation

- Establish a systematic approach for developing, evaluating, and implementing interventions for family caregivers^3^ (similar to the drug discovery and translation pipeline). Private/public partnerships should be pursued to foster the implementation of proven interventions as well as exploring how such interventions may fit within existing health care organizations such as Accountable Care Organizations, and/or existing programs, such as the NFCSP.^3^
- Consider establishing a national research institute for family caregiving or bolstering support for an existing caregiver institute that can take on this role (e.g., the Family Caregiver Alliance and the National Alliance for Caregiving).
- Develop a consensus-based national priority-driven family caregiver research agenda and outline the associated funding and resources required to operationalize the research. Examples of topics that should be considered for the priority research agenda are outlined in Table 2. The process to discern priorities should incorporate a critical appraisal of systematic reviews of caregiver interventions and explore the merit of multi versus single component.
- The Centers for Medicare and Medicaid Services should establish rigorous evaluation of metric-based family caregiver outcomes linked to health professional competence in family-centered care as standard practice.^3^
- Strengthen the development, implementation, and evaluation of technology to support caregivers through collaborative and innovative partnerships between researchers and the technology industry.^3^
- Foster the recognition of the services rendered to caregivers to improve routine data collection systems through amendments to Current Procedural Terminology or Healthcare Common Procedure Coding System.^3^
- Support and encourage the U.S. Department of Health and Human Services to establish a process for identifying, prioritizing, and harmonizing caregiver-related measures across sites and models of care.^3^
- Expand the data collection resources within the U.S. Departments of Health and Human Services, Labor, and Veterans Affairs to improve monitoring and reporting on the experience of family caregivers.^3^
- Collaborate with the NIH, and Patient Centered Outcomes Research Institute (PCORI) to establish a long-term funding stream for family caregiver research.^1^
- Develop a more systematic approach to involving consumers and caregiver organizations in the design and execution of research studies.^1^
- Seek support from The Secretaries of the U.S. Departments of Health and Human Services, Labor, and Veterans Affairs to work with leaders in health care and philanthropy to extend collaborative family caregiver research endeavors.^3^ Leverage work done by the National Palliative Care Research Center (acting in partnership with the Center to Advance Palliative Care), which provides a mechanism for establishing research priorities, preparing a new generation of researchers, and coordinating and supporting studies aimed at improving care (NPCRC, 2013). Seek support also from The Palliative Care Research Cooperative Group, which offers a mechanism for connecting researchers and clinicians across varied clinical settings and facilitating timely completion of complex studies, including randomized controlled trials, by pooling resources and expertise across sites.^1^
- Develop “online” systems that offer mixed methods of support (e.g., a virtual coach and/or telephone guidance) for caregivers who would typically not engage through this mode, coaching them through the experience. Although some family caregivers will not embrace “online” options and, therefore, require face-to-face intervention, it is important to invest in this approach as well, given economies of scale and efficiencies in operations.
- Additional program announcements from NIH, PCORI, and disease-specific foundations (e.g., American Cancer Society) targeting caregiver intervention research aligned with the priority-driven research agenda.
- Perform formal scoping review of novel clinical/service delivery family caregiver support initiatives. Evidence-based exemplars including veterans, Alzheimer's Association, and Family Caregiver Alliance could be systematically implemented nationally.
- Conduct a comprehensive review of the CARE Act to ascertain its impact and areas for improvement.

**Table 2.** Examples of Topics That Should Be Considered for the Priority Research Agenda

The impact and feasibility of sustaining interventions over time.^3^
Circumstances where multiple caregivers are involved (given that many families share responsibilities) and explore feasible means of support in these scenarios.^3^
Determining when to use dyadic interventions rather than caregiver(s) only.
Involvement of more diverse caregiver populations and vulnerable populations including (but not limited to) caregivers for Hispanic, African American, Native American, and other ethnic and racial groups; long-distance caregivers; and HIV/AIDs caregivers, LGBTQ caregivers, and caregivers for people with substance abuse.^3^
Exploration of the financial, health, and social services utilization impact of family caregiver interventions.^3^
Fostering family caregiver research in the community through extending and supporting practice-based research networks.^1^
Health care and social services workforce capacity issues (status and future forecasting) related to family caregiver support.
Compare and contrast the needs and experiences of family caregivers of people with serious illness who do and do not receive hospice social services.
Bereavement research including descriptions of issues, tools for assessment, and effective interventions.
Assess the bereavement services available to family caregivers of patients who were not enrolled in hospice.
Study family caregivers of patients with different diseases to compare and contrast needs.
Preventive interventions to foster the psychological well-being of family caregivers, including optimal ways for discerning caregivers “at risk.”
How patient/family caregiver dynamics impact upon clinical decisions made by doctors and nurses.
Development and testing of brief interventions targeting family caregivers that can be readily delivered in the practice setting and an associated triage system that links caregivers to various types of programs (high or low intensity) depending on their caregiver needs.
Potential benefits, utility, and resources required to establish a National Family Caregiver call center (phone and online) to support family caregivers of people with serious illness who need urgent advice (this process should incorporate a review of any existing services of this type).
Foster self-management and caregiver problem solving.
Conduct more prospective studies that clearly delineate the transition from disease management to supportive care to end-of-life care to better understand the caregiving trajectory and how these transitions affect the caregiver and formal care provided to the care recipient.^3^
Determine the core information that should be offered to all family caregivers about the common elements of the role and develop evidence-based processes for tailoring additional information according to need.
Ascertain specific types of respite that offer the best outcomes for caregivers, patients, and the health care and social services system.
Promote research investment and collaboration from federal and state agencies to explore the feasibility and impact of any changes to policy and financial aid options for caregivers.^3^
Determine the barriers and enablers to the uptake of web/e-health-based interventions.

#### Service delivery (clinical and social care) and accreditation

- Develop quality measures related to family caregiver support in accreditation organizations such as the National Committee for Quality Assurance.^3^
- Ensure that identification, screening, and caregiver assessment occur at each significant point (e.g., diagnosis of serious illness) in care delivery for the care recipient—including delivery of publicly funded long-term services and supports, annual wellness examinations, physician visits, admission and discharge for hospitals and emergency rooms, and chronic care coordination and care transition programs.^3^ This should be undertaken by a health coach/educator who can then involve others, for example, clinicians as pertinent.
- Accrediting and standard-setting organizations must incorporate evaluation of how well institutions assess and respond to family caregiver needs. The joint commission should ensure that surveyors are competent to assess family caregiver training and support. The National Quality Forum, which endorses national consensus standards for measuring and publicly reporting on performance, should explicitly develop standards that include the role of family caregivers and build on some of its existing family-centered initiatives and resources.^7^
- Government and accrediting bodies should incorporate psychosocial assessments of primary caregiver(s) into regulations and quality audits. These should include the caregiver's health and ability to provide care, willingness to provide care, and any special skills the caregiver may have. Some families have multiple caregivers so additional coordination may be needed.
- Request the Office of the National Coordinator for Health Information Technology (ONC) to create a certified health electronic record technology (CEHRT) standard for family caregiver assessment and associated care plan (regardless of the site of care).
- Review existing “care coordination” programs to ensure family caregivers are receiving adequate support.
- Expand and review the use of health coaches and/or health educators (in hospitals, in the community, and online) to help guide and support family caregivers. The health coaches would ideally represent the cultural and/or faith-based context of the caregiver and could work in partnership with medical/nursing providers and social services to be the main conduit for caregiver guidance and support. Potentially the service could be funded through the “Medicare Advantage” program.
- Expand the availability of community-based palliative care to increase accessibility for people with serious illness.
- Establish consensus and evidence-based guidelines for the appropriate timing of referrals to palliative care specialists (medicine, nursing, social work, and allied health) and the typical parameters/expectations of generalist health care providers in the provision of the core elements of palliative care (including family caregiver support).
- Create a standard that all health and social service workers involved in the care of people with serious illness receive training in palliative care (including family caregiver support).
- Increase the proportion of palliative care trained social workers, nursing assistants, and community health workers across settings.
- Ensure that hospitals develop systems for gathering information about the person with serious illness and their family caregiver from their community service providers so that information exchange is fostered between hospital and home and vice versa.
- Provide necessary coalition building within the community, such as socials services (nutrition, transportation, medical equipment, and respites services), ensuring collaboration with medical, nursing, social, cultural, and faith-based services that require a major cultural shift from a medical-/hospital-focused model.
- Implement suitably evidence-based psychosocial and/or behavioral family caregiver interventions as they were designed rather than adapting them to local contexts. Although often well intentioned, changing the delivery and/or dose of the intervention can have negative outcomes.
- Acknowledge in standards for family caregiver support that in certain circumstances family may not be the most appropriate choice of caregiver, requiring alternative solutions to be discerned.
- Expand accessibility and affordability of options for respite care.
- Appoint a designated worker (e.g., health coach/educator or care coordinator) to support family caregivers when a person is diagnosed with serious illness.
- Develop a regulation requirement to ensure that bereavement support is available for family caregivers.
- Conduct a systematic assessment of and provide preparatory training to the family caregiver before the patient's discharge from hospital or clinic.
- Provide incentives for greater integration of family caregivers in advance care planning with clinicians.
- Develop and implement specific strategies (including providing reimbursements) that will allow time for clinicians to be involved in family caregiver support planning processes.
- Incorporate family caregiver support strategies within long-term care facilities and nursing homes. Ideally, where pertinent, apply accreditation standards and processes similarly across care settings.
- Review remuneration/compensation provided to frontline workers (e.g., home health aides) who are frequently instrumental in supporting family caregivers and enabling people to remain at home for as long as feasible.
- Develop and disseminate a tool box of evidence-based family caregiver interventions with implementation manuals and materials to health care providers.
- Invest resources in the development of a multicultural workforce comprising local/community leaders who can influence change and increase uptake of family caregiver support options.
- Comprehensively explore the role of “volunteers” (lay people) who offer support to people diagnosed with serious illness and their family caregivers and assess their impact.
- Conduct a workforce analysis to determine status, projections, and pragmatic approach to family caregiver support. Specialist palliative care providers cannot be expected to meet the needs of all those affected by serious illness.
- Review published standards/guidelines for supporting family caregivers of people requiring palliative care^16,17^ for uptake and impact.
- Adhere to the National Standards for Culturally and Linguistically Appropriate Services in Health and Health Care to provide quality care that is effective, equitable, understandable, respectful, and responsive to caregivers' cultural health beliefs and practices, preferred languages, and health.^3^
- Structure care coordination services, particularly in new Medicare and Medicaid payment mechanisms designed to pay for it, to enable caregivers to access long-term services and other social supports through Area Agencies on Aging and other agencies^3^ including agencies for younger patients, patients with disabilities, professional disease-specific organizations such as ALS Society, Alzheimer's Association, American Heart Association, and the American Cancer Association.

#### Public engagement

- Launch a public campaign about the role and impact of being a family caregiver for a person living with a serious illness, and provide resources on how they can access support. This will require a well-funded, systematic, novel, and sustained communication strategy delivered through a variety of methods (potentially through direct consumer marketing as drug companies do) and designed by suitably qualified marketing advisers/consultants.
- Develop specific strategies to market the concept of serious illness and palliative care to various cultural and faith-based communities to foster trust in this type of care and allay misconceptions.
- Establish information pathways in health care settings that promote family caregivers' sense that they are worthy recipients of support.
- Provide information to employers that describes the implications of having employees who are family caregivers and clarify how helping these caregivers may in fact reduce costs for the employer.
- Dedicate more resources to marketing information about what palliative care is/is not (including how it differs from hospice/end-of-life care) to the public, policy makers, and health professionals.
- Create and disseminate clearer information to patients, family caregivers, and health professionals on HIPAA, including what it intends to accomplish and information sharing that is and is not allowed under the law.
- Focus on mobilizing human capital in the community, given limited funding and resources (which is unlikely to change). Invest in community engagement programs such as New York City's friendly visitor and bill payer programs.
- Promote public engagement campaigns that foster uptake and involvement of family caregivers in advance care planning.
- Civic leaders, public health, and other governmental agencies, community-based organizations, faith-based organizations, consumer groups, health care delivery organizations, payers, employers, and professional societies should engage their constituents and provide fact-based information through intergenerational education about care of people with advanced serious illness to encourage advance care planning and informed choice based on the needs and values of individuals.^1^
- Consider appointing a high-profile public figure to assist with lobbying and profiling family caregiver issues.

### Call to action: Strategy refinement and operationalization

**FIG. 1.**
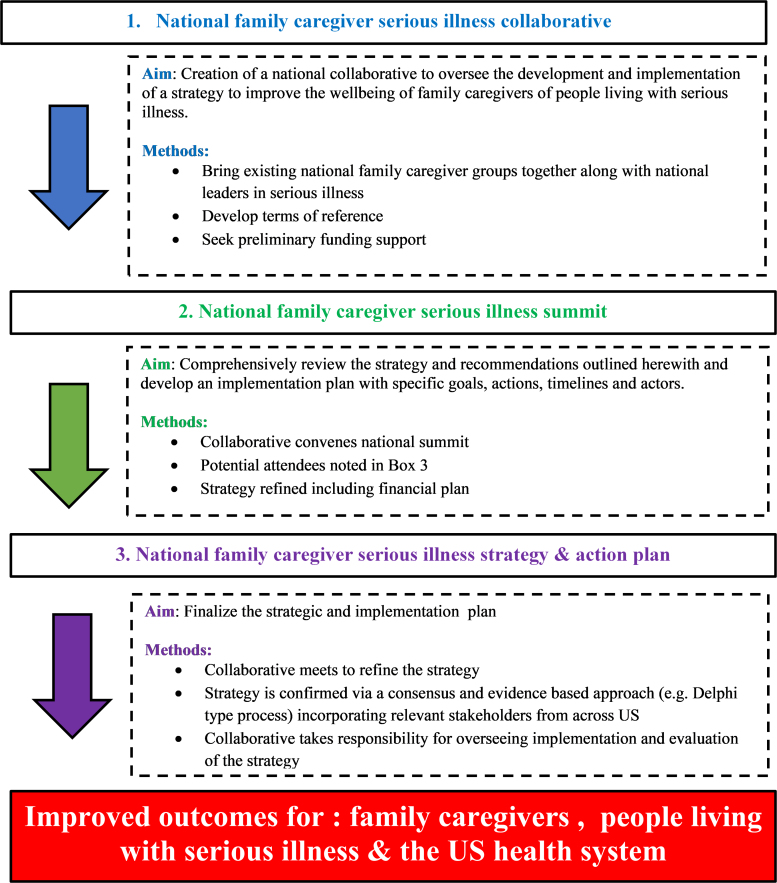
National family caregiver serious illness strategy and action plan.

For the strategy and its recommendations to be actioned, a National Caregiver Serious Illness Collaborative should be established. A coordinated effort is critical to improving support across the country.^18^ As outlined in Figure 1, it could take responsibility for progressing the refinement of these strategies and developing a specific operational plan that can be monitored and evaluated. This should include organizations such as the Family Caregiver Alliance, National Alliance for Caregiving, AARP, and Caregiver Action Network and incorporate those with significant experience in serious illness.

The collaborative should seek funding and resources to enable it to conduct its work. Philanthropic and/or corporate (e.g., multinational) institutions should be approached and consideration given to applying for a national grant (e.g., AARP, the Commonwealth fund). The collaborative should then convene a national family caregiver summit with key stakeholders (with options for “virtual” attendance/participation); attendance at the summit should be conditional upon willingness to take on responsibilities as pertinent after the summit. Proposed potential key stakeholders are outlined in Table 3.

**Table 3.** Examples of Key Stakeholders to Inform the Evolution and Implementation of the Strategy

Key stakeholder groups	Examples
Family caregiver and patient advocate organizations	Family Caregiver Alliance, National Alliance for Caregiving, AARP, Caregiver Action Network
Hospice and palliative care organizations	National Hospice and Palliative Care Organization, the American Academy of Hospice and Palliative Medicine, Social Work Hospice and Palliative Care Network, the National Association of Social Workers, CAPC, NPCRC, Palliative Care Research Cooperative, Association of Professional Chaplains, Health Care Chaplaincy Network, Hospice and Palliative Nurses Association and the National coalition of hospice and palliative care. Governmental health agencies such as the U.S. Departments of Health and Human Services, Labor, and Veterans Affairs, National Institute of Health, and other federal agencies
Disease-specific organizations and advocacy groups	Oncology Associations, Cancer Support Community, American Cancer Society, American Heart Association/American Stroke Association, Alzheimer's Association, Patient Quality of Life Coalition; American Society of Clinical Oncology, Oncology Nursing Society, American Geriatric Society, American Heart Association, American Stroke Association, ALS Society, Long-Term Care Association, Skilled Nursing Facility Associations, Hartford Institute for Geriatric Nursing
Allied partners	Technological companies, professional societies/colleges, employment and workforce planners, national curriculum planners, etc.

CAPC, Center to Advance Palliative Care; NPCRC, National Palliative Care Research Center.

The summit should focus on:

1. Comprehensively workshopping the strategy already outlined so that it can be refined and key tasks prioritized.
2. Developing an an implementation plan linked to the strategy that explicitly identifies timelines and “actors” responsible for specific actions.
3. Developing a strategy for financial support. In terms of funding and resourcing, strategic actions, changes in the public sector, and support and guidance of the private sector will be required. Employers of all types have a vested interest in supporting caregivers. The public sector cannot achieve all necessary progress on its own; a public–private innovation fund could leverage private funding to complement public resources and fill gaps in public funding.^3^ The fund, for example, could sponsor the development of market-driven approaches for lessening the strain of caregiving on families—targeting innovative services and products that are scalable and sustainable. Potential products include assistive technologies, remote monitoring and sensing systems, telehealth applications, and other tools to assist family caregivers and to enable older adults to continue living in their home and communities. These systems could also be linked to health care and social service providers to aid in care coordination efforts.^3^

After the summit, the caregiver collaborative should refine the strategic, implementation, and financial plan, seeking consensus and endorsement of the key stakeholders. This could be done through a Delphi-type survey. The outcomes of the summit should be formally reported and made public (journal publication, conferences, media, etc.). The caregiver collaborative should ensure it is represented by an appropriate number of active and past caregivers.

### Limitations

The strategy outlined herewith should be viewed as an initial phase as part of a longer term process to systematically improve caregiver support. Owing to limited resources and time, engagement across all key stakeholders and U.S. states was not feasible. Nor was it possible to prioritize recommendations and identify actors to take responsibility for relevant tasks. As outlined, subsequent study will be needed on prioritization of tasks (including identifying actors responsible), operationalization, and implementation of the strategy. Furthermore, important next steps will be to prioritize the policy agenda and other related activities for specific caregiver cohorts within the seriously illness purview.^5^

## Conclusions

The urgent need for a systematic approach to comprehensively improve support for family caregivers of people with serious illness in the United States is clear, given the current state that has recently been highlighted as a public health crisis.^18^ The economic implications should not be understated given the large societal burden resulting from reduced work productivity and increased use of health care resources by caregivers.^19^

Progressing the strategic directions developed in this plan will require a commitment from federal and state governments to implement and monitor progress toward the vision of improved quality of life for caregivers. The success of this strategy will also depend upon leaders from key stakeholder groups working collaboratively to reach consensus on priorities and a willingness to accept responsibility for completing tasks effectively.

The markers of a transformed system will result in a society in which family caregivers have their own health and well-being considered, together with their rights and protections. They would also have access to evidence-based health information and support when they need it. Providers would have the competencies to provide high-quality (best practice), culturally appropriate, and family-centered services.^3^

## Abbreviations Used

ADRC: Aging and Disability Resource Center
CARE: Caregiver Advise, Record, Enable
HIPAA: Health Insurance Portability and Accountability Act
NIH: National Institute of Health
PCORI: Patient Centered Outcomes Research Institute
NFCSP: The National Family Caregiver Support Program
